# An Accuracy Comparison of Micromechanics Models of Particulate Composites against Microstructure-Free Finite Element Modeling

**DOI:** 10.3390/ma15114021

**Published:** 2022-06-06

**Authors:** Yunhua Luo

**Affiliations:** Department of Mechanical Engineering, University of Manitoba, Winnipeg, MB R3T 2N2, Canada; yunhua.luo@umanitoba.ca

**Keywords:** particulate composite, representative volume element, effective property, contrast of phase properties, phase volume fraction, microstructure-free finite element modeling

## Abstract

Micromechanics models of composite materials are preferred in the analysis and design of composites for their high computational efficiency. However, the accuracy of the micromechanics models varies widely, depending on the volume fraction of inclusions and the contrast of phase properties, which have not been thoroughly studied, primarily due to the lack of complete and representative experimental data. The recently developed microstructure-free finite element modeling (MF-FEM) is based on the fact that, for a particulate-reinforced composite, if the characteristic size of the inclusions is much smaller than the composite representative volume element (RVE), the elastic properties of the RVE are independent of inclusion shape and size. MF-FEM has a number of advantages over the conventional microstructure-based finite element modeling. MF-FEM predictions have good to excellent agreement with the reported experiment results. In this study, predictions produced by MF-FEM are used in replace of experimental data to compare the accuracy of selected micromechanics models of particulate composites. The results indicate that, only if both the contrasts in phase Young’s moduli and phase Poisson’s ratios are small, the micromechanics models are able to produce accurate predictions. In other cases, they are more or less inaccurate. This study may serve as a guide for the appropriate use of the micromechanics models.

## 1. Introduction

The analytical or semi-analytical solutions developed from micromechanics models of composite materials provide an efficient method for the prediction of effective elastic properties. Compared with the experimental methods and the computer modeling approaches, micromechanics-based analytical solutions are preferred by material engineers in the analysis and design of new composites. The design process is usually an iterative process and often requires a large number of elasticity characterizations for the intermediate designs, which would be very costly and time-consuming if the experimental methods or computer modeling were to be used. Most micromechanics models have been developed for particulate or short-fiber reinforced composites [1,2,3,4,5,6], primarily due to their wide applications in engineering structures and industrial products. These micromechanics models are also important for the description of mechanical behavior of functionally graded materials and structures [7,8]. However, the accuracy of these micromechanics models varies widely, and it is affected by factors such as the volume fraction of inclusions and the contrast of phase properties. Information regarding the accuracy variation of the micromechanics models is necessary for material engineers to appropriately apply them in composite analysis and design. However, a thorough study on the accuracy variation of micromechanics models has not yet been conducted, primarily due to the lack of complete and representative experimental data. Although mechanical testing is the ideal method for evaluating the accuracy of micromechanics models, mechanical testing is expensive and time-consuming. Experimental data reported in the literature do not cover the entire range of inclusion volume-fraction and are unable to represent the various contrasts of phase properties, e.g., [9,10,11] among others. Furthermore, experimental data are often adversely affected by factors such as experimental setup, defects in test specimens, and random measurement errors. 

Conventional microstructure-based finite element modeling (MB-FEM) is a more efficient approach than the experimental methods, but it has a number of deficiencies. For example, it is time-consuming to generate a workable geometric model for the composite microstructure; it is challenging to achieve a high volume-fraction of inclusions; and the quality of the finite element mesh is affected by the geometric shape of inclusions and the minimum distance between neighboring inclusions. The recently developed microstructure-free finite element modeling (MF-FEM) [12] may eliminate these deficiencies. It has been demonstrated that the elastic properties predicted by the MF-FEM have good to excellent agreement with the reported experimental data [12]. 

In this paper, predictions of MF-FEM are used in replacement of experimental data to study how the accuracy of micromechanics models varies with the volume fraction of inclusions and the contrasts of phase properties. The objective of this study is to provide a guide for the appropriate use of the micromechanics models. 

## 2. Materials and Methods

Elastic properties of representative two-phase composites are predicted by the MF-FEM, which are used as references in the comparison of selected micromechanics models. The four particulate composites described in Table 1 are used for the study. They represent different natural or engineering composite materials, with typical combinations of contrasts in the phase Young’s moduli and phase Poisson’s ratios. Generally, a phase material that has larger Young’s modulus would have smaller Poisson’s ratio, and vice versa. The contrast of a phase property is measured by the ratio of the larger value to the smaller one. 

The elasticity of a composite material and its constituents are often described by four elastic constants, i.e., Young’s modulus, shear modulus, bulk modulus, and Poisson’s ratio. These properties are not independent from each other but are related via the general elasticity relations. If two of them are known, the rest can be determined from the elasticity relations. For the above reason, the MF-FEM and the selected micromechanics models are required to predict at least two of the four elastic properties. 

For consistency, the symbols listed in Table 2 are used in the description of elastic properties of composites and their phases.

### 2.1. Microstructure-Free Finite Element Modeling (MF-FEM) of Composite Representative Volume Element (RVE)

**Figure 1:**
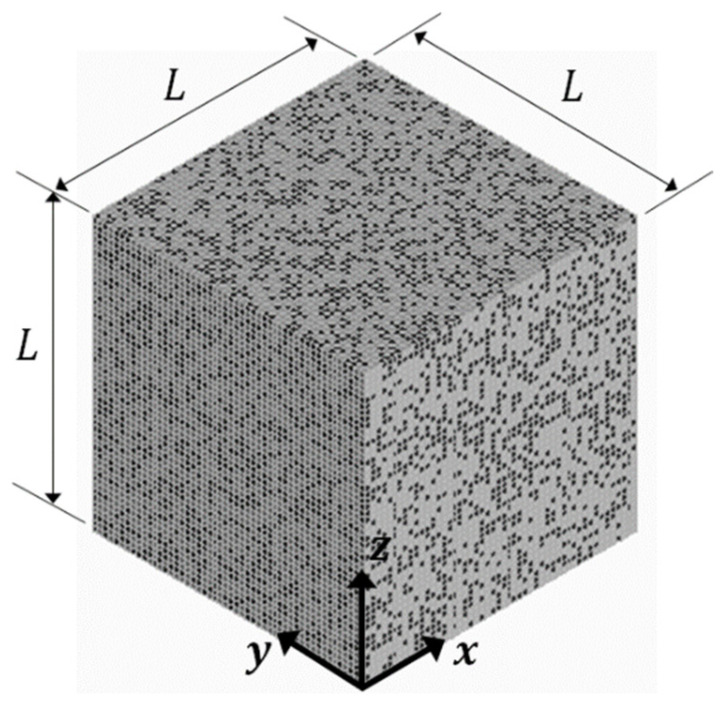
Representative volume element used in MF-FEM.

Microstructure-free finite element modeling (MF-FEM) is a recently developed approach for the study of particulate composites [12]. The fundamental difference between the MF-FEM and the conventional microstructure-based finite element modeling (MB-FEM) is in the way that the inclusions are represented in the finite element model. In the MB-FEM, a geometric model must be constructed to describe the composite microstructure that consists of a matrix and inclusions, which involves a great amount of work but it is not necessary. Based on the continuum mechanics of composites [13,14,15], for a particulate composite, if (1) the inclusions have small aspect ratio, (2) their characteristic size is much smaller than the representative volume element (RVE), and (3) the inclusions have a statistically uniform random distribution in the RVE, the elastic properties of the RVE are independent of the shape and size of the inclusions. The above assumptions are also adopted in almost all the micromechanics models of particulate composites. Previous studies [5,16,17,18] indicate that if the maximum characteristic size of inclusions is smaller than 0.04 times of the RVE dimensions, the RVE elastic properties are almost independent of inclusion shape and size. Now that the shape and size of small inclusions have no effect, the MF-FEM use elements to represent the inclusions. In the version of MF-FEM implemented previously [12], first a uniform mesh of brick elements is generated for the RVE that contains no inclusion; then, a number of the elements are randomly selected and assigned with the inclusion properties, and the rest of the elements are set to have the matrix properties. There are different methods to implement the random selection of elements to represent inclusions. In this study, the MATLAB function p = randperm(N) is used to generate a random permutation of the integers from 1 to N without repeating numbers, where N is the total number of elements in the RVE finite element mesh; the first n elements are selected as the inclusions, the total volume of the selected n elements is required equal to the volume of inclusions, the latter is calculated from the desired volume-fraction of inclusions. No periodicity is required in the distribution of inclusions. One sample of RVE constructed in such a way is displayed in Figure 1. 

The RVE is in a cubic shape and the dimensionless side length is L=100, and the element size is 2. Therefore, the size ratio between the elements and the RVE is 1:50, which is smaller than the threshold, such that the shape and size of inclusions have no effect on the elastic properties of the RVE. The task of the MF-FEM is to characterize the effective Young’s modulus and effective Poisson’s ratio of the RVE in the three axial directions displayed in Figure 1. 

Theoretically, if the assumptions described in the above are satisfied, the composite RVE should be isotropic in its elastic properties. However, the actual distribution of inclusions may not be strictly random and uniform. Therefore, the RVE properties may still be slightly anisotropic [12]. To eliminate the residual anisotropy, the properties in the x, y, and z directions are averaged. The boundary conditions used in the characterization of RVE properties are listed in Table 3. 

Based on the mean-field homogenization theory [19,20], the effective Young’s moduli (${\bar{E}}_{i}$) and effective Poisson’s ratios (${\bar{\nu }}_{ij}$) of the RVE in the three axial directions are determined from the average stresses (${\bar{\sigma }}_{i}$) and average strains (${\bar{\varepsilon }}_{i}$), i.e.,

(1) $${\bar{E}}_{i}=\frac{{\bar{\sigma }}_{i}}{{\bar{\varepsilon }}_{i}}\;,\;(i=x,y,z)$$

(2) $${\bar{\nu }}_{ij}=-\frac{{\bar{\varepsilon }}_{j}}{{\bar{\varepsilon }}_{i}}\;,\;(i,j=x,y,z)$$

The average stresses and strains are calculated from the finite element stresses ($\sigma_{i}$) and strains ($\varepsilon_{i}$) via

(3) $${\bar{\sigma }}_{i}=\frac{1}{V}\int_{V}\sigma_{i}\;dV,\;\;\;{\bar{\varepsilon }}_{i}=\frac{1}{V}\int_{V}\varepsilon_{i}\;dV\;(i=x,y,z)$$

where V is the volume of the RVE.

The effective Young’s modulus and effective Poisson’s ratio of the RVE are obtained as

(4) $$\bar{E}=\frac{{\bar{E}}_{x}+{\bar{E}}_{y}+{\bar{E}}_{z}}{3}$$

(5) $$\bar{\nu }=\frac{{\bar{\nu }}_{xy}+{\bar{\nu }}_{yx}+{\bar{\nu }}_{yz}+{\bar{\nu }}_{zy}+{\bar{\nu }}_{zx}+{\bar{\nu }}_{xz}}{6}$$

### 2.2. The Selected Micromechanics Models of Particulate Composites

A large number of micromechanics models have been developed for the prediction of composite elastic properties [3,5,6] but not all of them will be compared in this study, due to the capability of the MF-FEM. The selected micromechanics models should satisfy the following criteria:

1. The micromechanics model was explicitly developed for particulate or short-fiber reinforced composites, where the composites can be considered a homogeneous and isotropic material at the length scale of the RVE.
2. The micromechanics model produces explicit analytical solutions for at least two of the four elastic properties.
3. The analytical solutions do not require an empirical coefficient.

Based on the above criteria, seven micromechanics models were selected. They are the Voigt and Reuss bounds, the Hashin–Shtrikman bounds, the Voigt–Reuss–Hill average, the Mori–Tanaka method, the generalized self-consistent scheme, the isotropized Voigt–Reuss model, and the product of exponential functions. To be referred later in this study, the analytical solutions produced by the selected micromechanics models are presented below with the symbols listed in Table 2.

 *(1)* *The Voigt and Reuss (VR) bounds [21,22]*

The Voigt and Reuss bounds are also referred to as the rule of mixtures and the inverse rule of mixtures in the literature when they are applied to predict the elastic properties of unidirectional fiber-reinforced composites. They are included in this comparison study due to their capability of providing the upper and lower bounds for the elastic properties of particulate composites. The Voigt and Reuss (VR) formulas can be generically presented as

(6a) $${\bar{P}}_{V}=f_{1}P_{1}+f_{2}P_{2}$$

(6b) $${\bar{P}}_{R}=\frac{P_{1}\;P_{2}}{f_{1}P_{2}+f_{2}P_{1}}$$

where, P and $\bar{P}$ represent a generic elastic property, which can be any one of the four elasticity constants; the subscripts V and R indicate Voigt and Reuss models, respectively. The formulas are often used to estimate bounds for Young’s modulus, sometimes also for shear modulus, Poisson’s ratio, and bulk modulus.

 *(2)* *The Hashin-Shtrikman (HS) bounds [23]*

The Hashin-Shtrikman (HS) bound formulas in Equation (7a–d) are derived from the variational principle of minimum potential energy with the concept of stress polarization, which have a stricter mathematical base than the VR bounds.

(7a) $${\bar{K}}_{L}=K_{1}+\alpha_{1},\;\alpha_{1}=\frac{f_{2}\left(K_{2}-K_{1}\right)\left(3K_{1}+4\;G_{1}\right)}{3f_{2}K_{1}+3\;f_{1}K_{2}+4G_{1}}$$

(7b) $${\bar{K}}_{U}=K_{2}-\alpha_{2},\;\alpha_{2}=\frac{f_{1}\left(K_{2}-K_{1}\right)\left(3K_{2}+4G_{2}\right)}{3f_{1}K_{2}+3f_{2}K_{1}+4G_{2}}$$

(7c) $${\bar{G}}_{L}=G_{1}+\beta_{1},\;\beta_{1}=\frac{5f_{2}\left(G_{2}-G_{1}\right)G_{1}\left(3K_{1}+4G_{1}\right)}{\left(12f_{2}+8\right){\left(G_{1}\right)}^{2}+\left(12f_{1}G_{2}+\left(6f_{2}+9\right)K_{1}\right)G_{1}+6G_{2}K_{1}f_{1}}$$

(7d) $${\bar{G}}_{U}=G_{2}-\beta_{2},\;\beta_{2}=\frac{5f_{1}\left(G_{2}-G_{1}\right)G_{2}\left(3K_{2}+4G_{2}\right)}{\left(12f_{1}+8\right){\left(G_{2}\right)}^{2}+\left(12f_{2}G_{1}+\left(6f_{1}+9\right)K_{2}\right)G_{2}+6G_{1}K_{2}f_{2}}$$

The subscripts, L and U, represent the lower and the upper bound of effective bulk modulus ($\bar{K})$ or effective shear modulus ($\bar{G}$).

 *(3)* *The Voigt–Reuss–Hill (VRH) average [24]*

The Voigt–Reuss–Hill average in Equation (8) provides a simple way to estimate the elastic constants of particulate composites.

(8) $$\bar{P}=\frac{{\bar{P}}_{V}+{\bar{P}}_{R}}{2}$$

where ${\bar{P}}_{V}$ and ${\bar{P}}_{R}$ are determined by Equation (6a,b).

 *(4)* *The Mori–Tanaka (MT) model [25]*

The Mori–Tanaka model is based on Eshelby’s elasticity theory for inhomogeneity in an infinite medium [20,26]. The average internal stress in the matrix is calculated with the transformation of the strain. Interaction among inclusions and the presence of a free boundary are considered in the average elastic energy. The MT formulas for the determination of effective shear modulus ($\bar{G}$) and effective bulk modulus ($\bar{K}$) are presented in Equation (9a,b).

(9a) $$\bar{G}=G_{1}+\frac{f_{2}\left(G_{1}-G_{1}\right)}{1+\frac{f_{1}\left(G_{2}-G_{1}\right)}{G_{1}+\frac{G_{1}\left(9K_{1}+8G_{1}\right)}{6\left(K_{1}+2G_{1}\right)}}}$$

(9b) $$\bar{K}=K_{1}+\frac{f_{2}\left(K_{2}-K_{1}\right)}{1+\frac{f_{1}\left(K_{2}-K_{1}\right)}{K_{1}+\frac{4}{3}G_{1}}}$$

 *(5)* *The Generalized self-consistent (GSC) model [3]*

In the generalized self-consistent model, a spherical inclusion is embedded in a concentric spherical annulus of the matrix material, which is in turn embedded in an infinite medium of unknown effective properties. This three-phase model is an improved version of the original tow-phase self-consistent model [27], where the spherical inclusion is directly embedded in the infinite medium of unknown effective properties. Although the concept is simple, the obtained analytical solutions are complex. The effective bulk modulus in Equation (10b) has the same expression as that in Equation (9b) of the MT model, but the solution of effective shear modulus is much more complex, as displayed in Equation (10a).

(10a) $$\bar{G}=\left(\frac{-B+\sqrt{B^{2}-A\cdot C}}{A}\right)G_{1}$$

(10b) $$\bar{K}=K_{1}+\frac{f_{2}\left(K_{2}-K_{1}\right)}{1+\frac{f_{1}\left(K_{2}-K_{1}\right)}{K_{1}+\frac{4}{3}G_{1}}}$$

The three coefficients A, B, and C in Equation (10a) are expressed by the phase shear moduli ($G_{1}$, $G_{2}$), phase Poisson’s ratios ($\nu_{1}$, $\nu_{2}$), phase volume fractions ($f_{1}$, $f_{2}$), and another three coefficients ($\eta_{1}$, $\eta_{2}$ and $\eta_{3}$).

$$\;\;A=8\left(\frac{G_{2}}{G_{1}}-1\right)\left(4-5\nu_{1}\right)\eta_{1}f_{2}^{\frac{10}{3}}-2\left(63\left(\frac{G_{2}}{G1}-1\right)\eta_{2}+2\eta_{1}\eta_{3}\right)f_{2}^{\frac{7}{3}}+252\left(\frac{G_{2}}{G_{1}}-1\right)\eta_{2}f_{2}^{\frac{5}{3}}-50\left(\frac{G_{2}}{G_{1}}-1\right)\left(7-12\nu_{1}+8\nu_{1}^{2}\right)\eta_{2}f_{2}+4\left(7-10\nu_{1}\right)\eta_{2}\eta_{3}\;B=-2\left(\frac{G_{2}}{G_{1}}-1\right)\left(1-5\nu_{1}\right)\eta_{1}f_{2}^{\frac{10}{3}}+2\left(63\left(\frac{G_{2}}{G_{1}}-1\right)\eta_{2}+2\eta_{1}\eta_{3}\right)f_{2}^{\frac{7}{3}}-252\left(\frac{G_{2}}{G_{1}}-1\right)\eta_{2}f_{2}^{\frac{5}{3}}+75\left(\frac{G_{2}}{G_{1}}-1\right)\left(3-\nu_{1}\right)\eta_{2}\nu_{1}f_{2}+\frac{3}{2}\left(15\nu_{1}-7\right)\eta_{2}\eta_{3}\;C=4\left(\frac{G_{2}}{G_{1}}-1\right)\left(5\nu_{1}-7\right)\eta_{1}f_{2}^{\frac{10}{3}}-2\left(63\left(\frac{G_{2}}{G_{1}}-1\right)\eta_{2}+2\eta_{1}\eta_{3}\right)f_{2}^{\frac{7}{3}}+252\left(\frac{G_{2}}{G_{1}}-1\right)\eta_{2}f_{2}^{\frac{5}{3}}+25\left(\frac{G_{2}}{G_{1}}-1\right)\left(\nu_{1}^{2}-7\right)\eta_{2}f_{2}-\left(7+5\nu_{1}\right)\eta_{2}\eta_{3}$$

The expressions of $\eta_{1}$, $\eta_{2}$, and $\eta_{3}$ are provided below.

$$\eta_{1}=\left(\frac{G_{2}}{G_{1}}-1\right)\left(7-10\nu_{1}\right)\left(7+5\nu_{2}\right)+105\left(\nu_{2}-\nu_{1}\right)\;\eta_{2}=\left(\frac{G_{2}}{G_{1}}-1\right)\left(7+5\nu_{2}\right)+35\left(1-\nu_{2}\right)\;\eta_{3}=\left(\frac{G_{2}}{G_{1}}-1\right)\left(8-10\nu_{1}\right)+15\left(1-\nu_{1}\right)$$

 *(6)* *The Isotropized Voigt-Reuss (Iso-VR) model [28]*

The Iso-VR model is the result of isotropization of the conventional Voigt and Reuss model, which together represent a transversely isotropic material model. The isotropization assumes that the normal and the shear strain energy in the transversely isotropic model are respectively equivalent to those in the isotropized model. The resulting formula is provided in the equation below.

(11) $$\bar{P}=\frac{2}{\frac{1}{{\bar{P}}_{V}}+\frac{1}{{\bar{P}}_{R}}}$$

The above formula is applicable to effective Young’s modulus and effective shear modulus [28].

 *(7)* *The product of exponential functions (PEF) [29]*

The product of exponential functions in Equation (12) was established from experimental data of Young’s moduli measured from bovine bone specimens. The formula is also applicable to effective shear modulus.

(12) $$\bar{P}=P_{1}^{f_{1}}\cdot P_{2}^{f_{2}}$$

In the above equation, $P_{1}$ and $P_{2}$ are originally the Young’s moduli of the organic and the inorganic phase in the bone. Bone is a typical composite material in nature, the formula is thus included in this comparison study. 

Two of the four effective properties that are predicted by the MF-FEM and by each of the selected micromechanics models are marked in Table 4. The rest of effective properties are determined via the elasticity relations. 

In this comparison study, the predictions of MF-FEM serve as the references in replace of experimental data to compare the accuracy of the selected micromechanics models. For the micromechanics models that predict specific values of the effective properties, the accuracy is measured by the relative error defined in Equation (13).

(13) $$\delta =\frac{\left|{\bar{P}}_{\mathrm{MF}-\mathrm{FEM}}-{\bar{P}}_{\mathrm{MM}}\right|}{{\bar{P}}_{\mathrm{MF}-\mathrm{FEM}}}\times 100\%$$

where ${\bar{P}}_{\mathrm{MF}-\mathrm{FEM}}$ and ${\bar{P}}_{\mathrm{MM}}$ are, respectively, the effective property predicted by the MF-FEM and one of the selected micromechanics models.

For the micromechanics models that estimate bounds, i.e., VR and HS, their performance is evaluated by how well the MF-FEM predictions are enclosed within the estimated bounds.

## 3. Results

**Figure A1:**
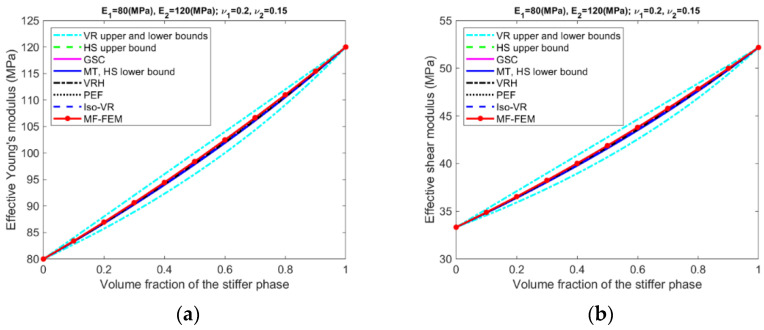

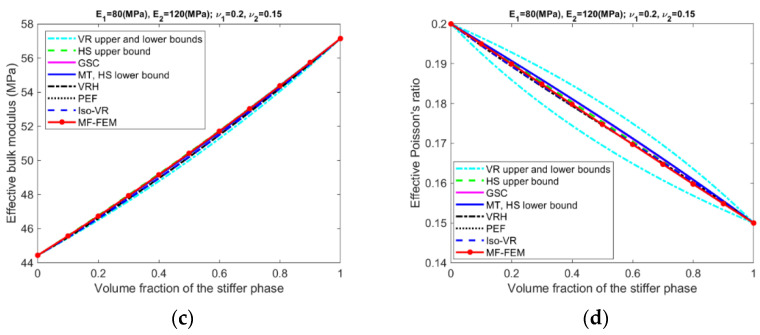
Composite #1—small contrast in both phase Young’s modulus and phase Poisson’s ratio. (**a**) Young’s modulus; (**b**) shear modulus; (**c**) bulk modulus; (**d**) Poisson’s ratio.

**Figure A2:**
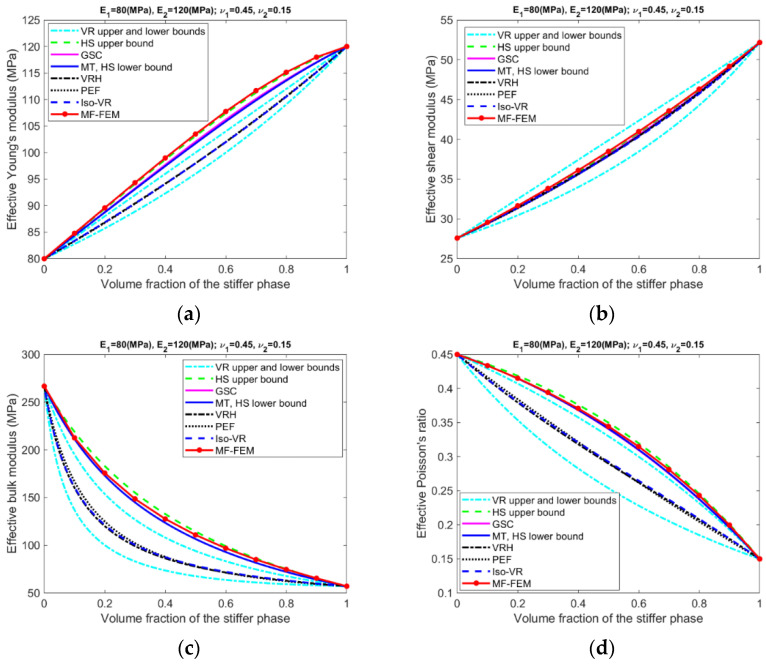
Composite #2—small contrast in phase Young’s modulus and large contrast in phase Poisson’s ratio. (**a**) Young’s modulus; (**b**) shear modulus; (**c**) bulk modulus; (**d**) Poisson’s ratio.

**Figure A3:**
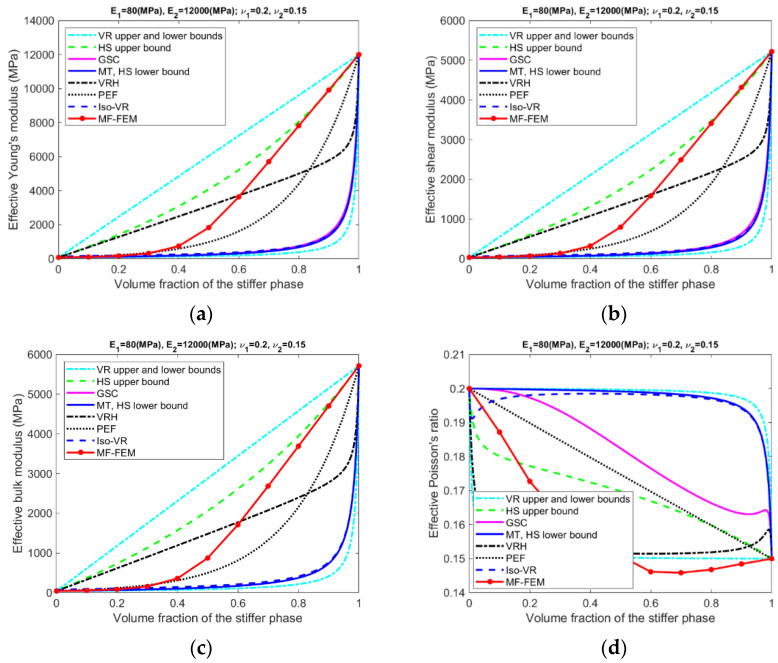
Composite #3—large contrast in phase Young’s modulus and small contrast in phase Poisson’s ratio. (**a**) Young’s modulus; (**b**) shear modulus; (**c**) bulk modulus; (**d**) Poisson’s ratio.

**Figure A4:**
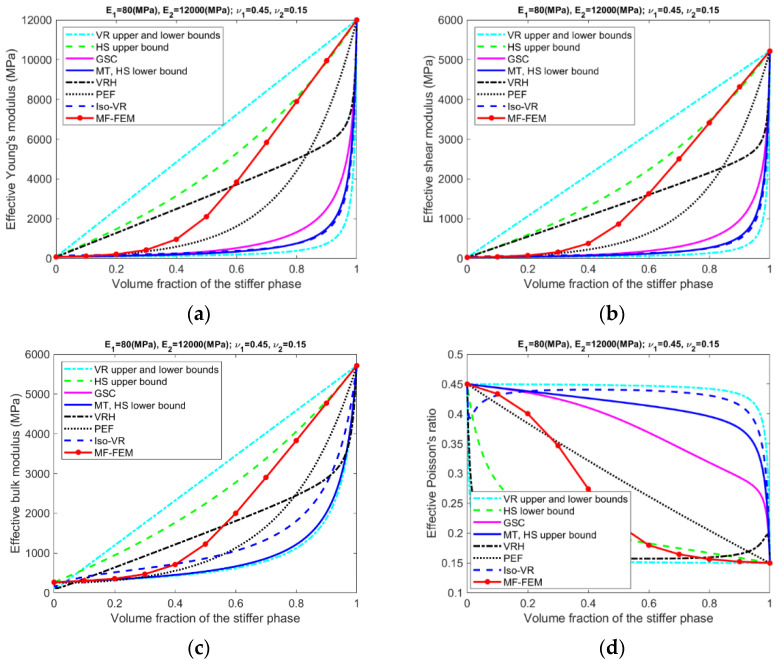
Composite #4—large contrast in both phase Young’s modulus and phase Poisson’s ratio. (**a**) Young’s modulus; (**b**) shear modulus; (**c**) bulk modulus; (**d**) Poisson’s ratio.

**Figure A5:**
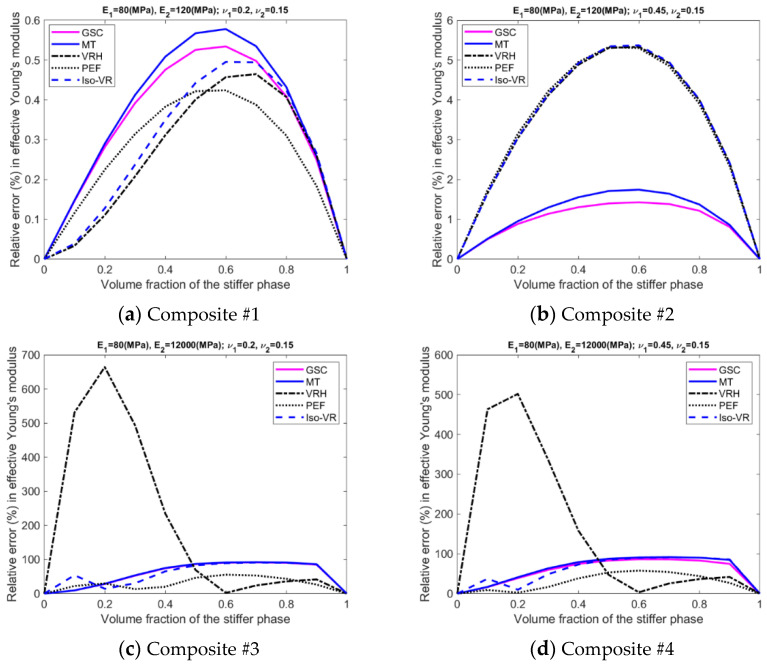
Relative error in effective Young’s modulus.

**Figure A6:**
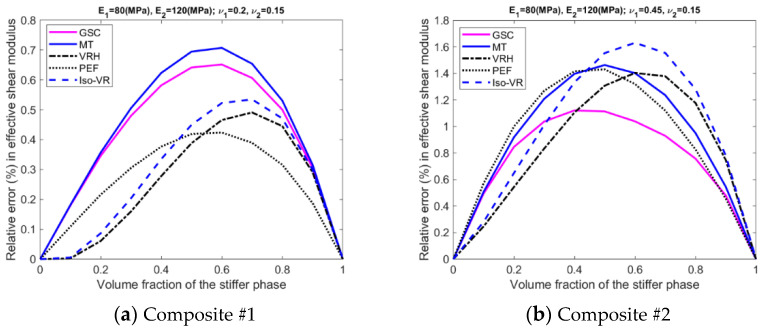

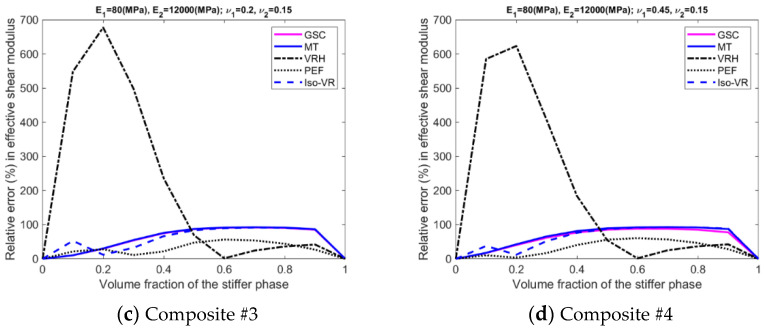
Relative error in effective shear modulus.

**Figure A7:**
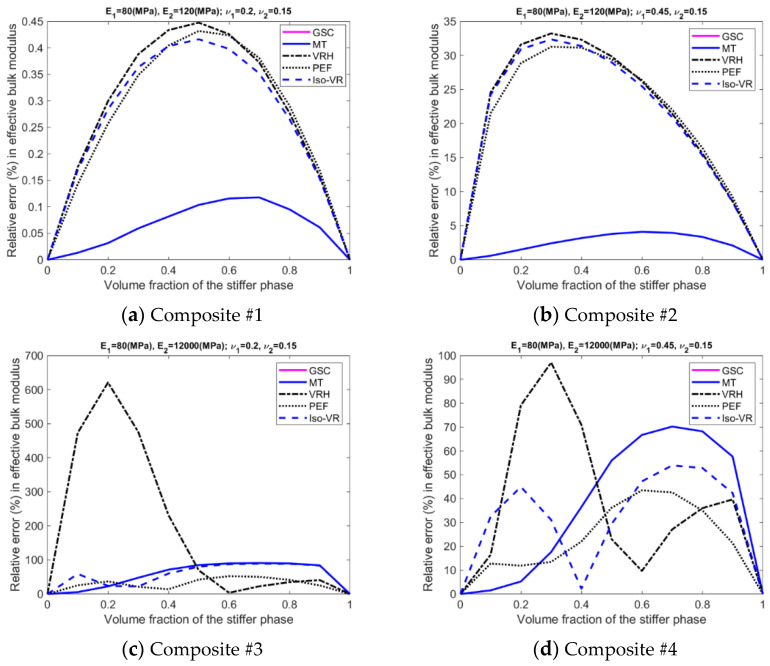
Relative error in effective bulk modulus. [Note: the pink line is coincident with the blue one].

**Figure A8:**
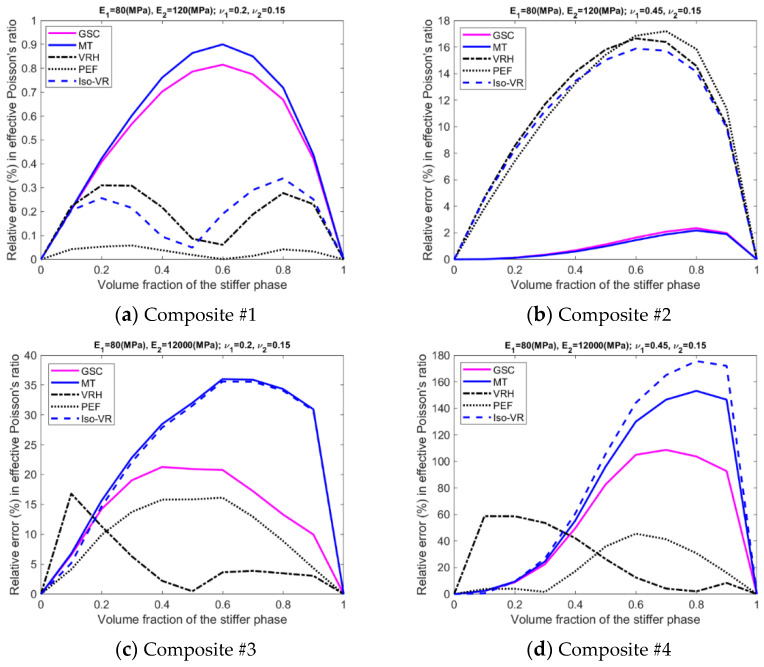
Relative error in effective Poisson’s ratio.

Figure A1, Figure A2, Figure A3 and Figure A4 in the Appendix A present the effective properties of the composites in Table 1, predicted by the MF-FEM and the selected micromechanics models with the volume fraction of the stiffer varying from 0.0 to 1.0. Based on the above results, relative errors in the effective properties predicted by VRH, GSC, MT, Iso-VR, and PEF are calculated using Equation (13). The obtained relative errors are displayed in Figure A5, Figure A6, Figure A7 and Figure A8 in the Appendix A. The relative errors are inhomogeneous and demonstrate fluctuation over the range of volume fraction; it is inconvenient to use them to measure the overall accuracy. Therefore, averages of the errors, calculated using the following equation, are used to measure the overall performance of the models.

(14) $$\bar{\delta }=\frac{\sum_{i}^{n}\delta_{i}}{n}$$

where i indicates one of the volume fractions $f_{2}=\left\{0.1,\;0.2,\;\cdots ,\;0.9\right\}$ and n=9.

**Figure 2:**
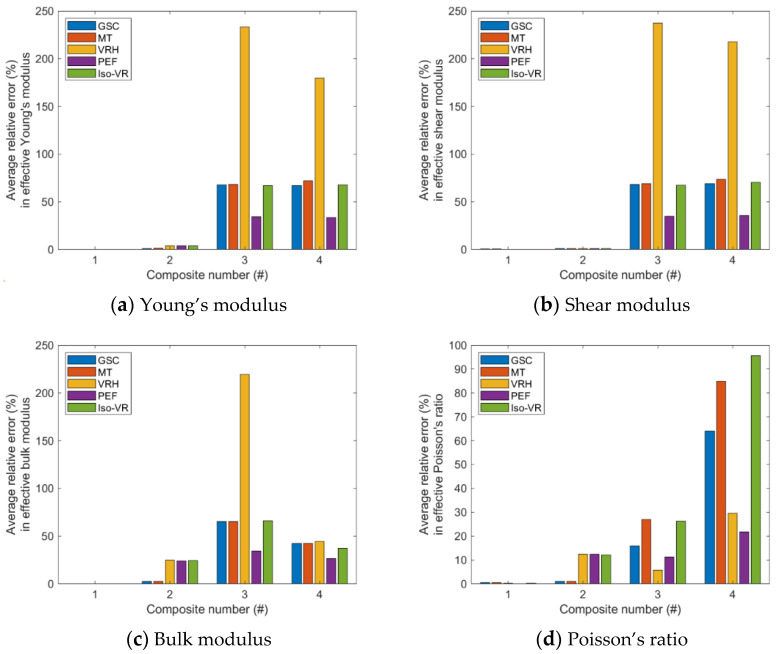
The average relative errors in the effective elastic properties.

The average relative errors in the effective Young’s modulus, shear modulus, bulk modulus, and Poisson’s ratio of the four composites (Table 1) are displayed in Figure 2.

The following observations can be made from the results regarding the accuracy of the five models, i.e., VRH, GSC, MT, Iso-VR, and PEF.

(1) The accuracy of the models is inhomogeneous over the range of volume fraction; see Figure A5, Figure A6, Figure A7 and Figure A8 in the Appendix A. For GSC and MT models, the maximum relative error usually occurs in the second half of the range. For VRH, Iso-VR, and PEF, the accuracy fluctuates over the range.
(2) Only for Composite #1, which has small contrasts in both its phase Young’s moduli and phase Poisson’s ratios, all the models have reasonable accuracy in all four effective properties. The maximum relative error is below 1%; see Figure A5a, Figure A6a, Figure A7a and Figure A8a, in addition to Figure 2.
(3) For Composite #2, which has a small contrast of phase Young’s moduli but a large contrast in phase Poisson’s ratios, the models have acceptable accuracy for effective Young’s modulus and effective shear modulus, with the maximum relative error below 6%. But the accuracy for effective bulk modulus and effective Poisson’s ratio is quite poor, with average error above 15%.
(4) For Composites #3 and #4, both have a large contrast in phase Young’s moduli, only if the volume fraction of the stiffer phase is low (<0.2), the models have acceptable accuracy, cf. Figure A1, Figure A2, Figure A3 and Figure A4. None of the models have acceptable overall accuracy, see Figure 2.
(5) Generally, PEF has better performance than the other models; see Figure A5, Figure A6, Figure A7 and Figure A8 and Figure 2.

The above observations indicate that contrasts in both phase Young’s moduli and phase Poisson’s ratios affect the accuracy of the models, but the effect from phase Young’s modulus is dominant. 

**Figure 3:**
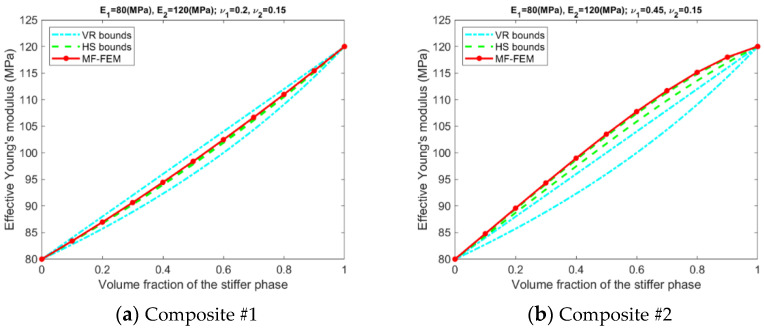

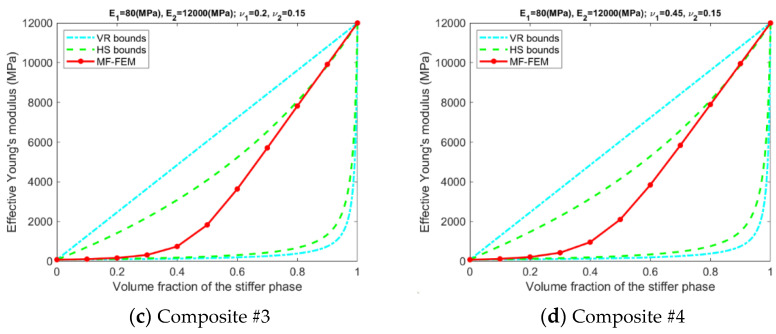
MF-FEM predictions and VR/HS bounds of effective Young’s modulus.

**Figure 4:**
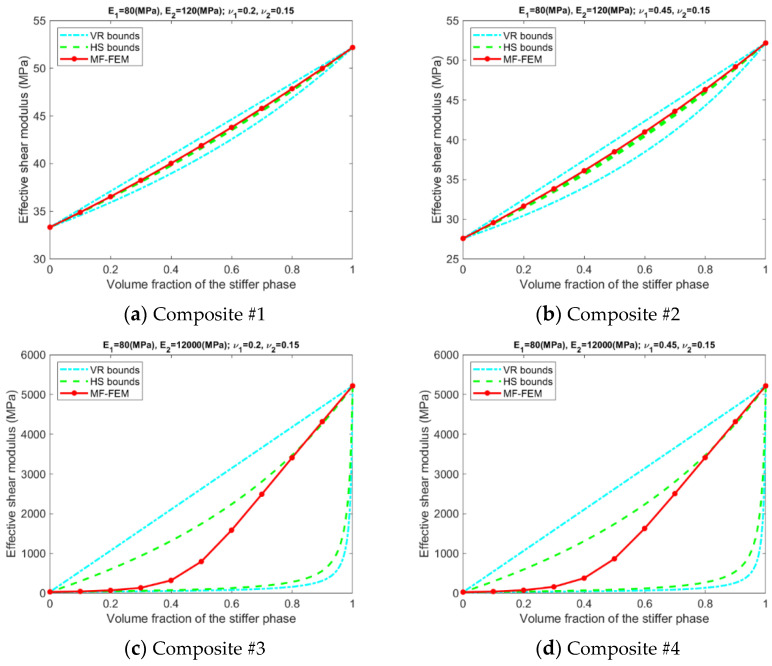
Relation between MF-FEM predictions and VR/HS bounds of effective shear modulus.

**Figure 5:**
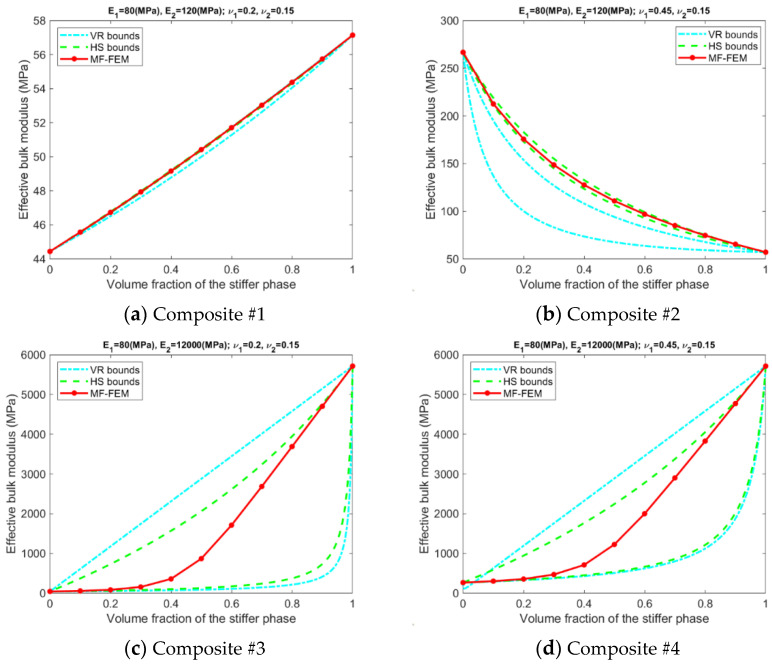
Relation between MF-FEM predictions and VR/HS bounds of effective bulk modulus.

**Figure 6:**
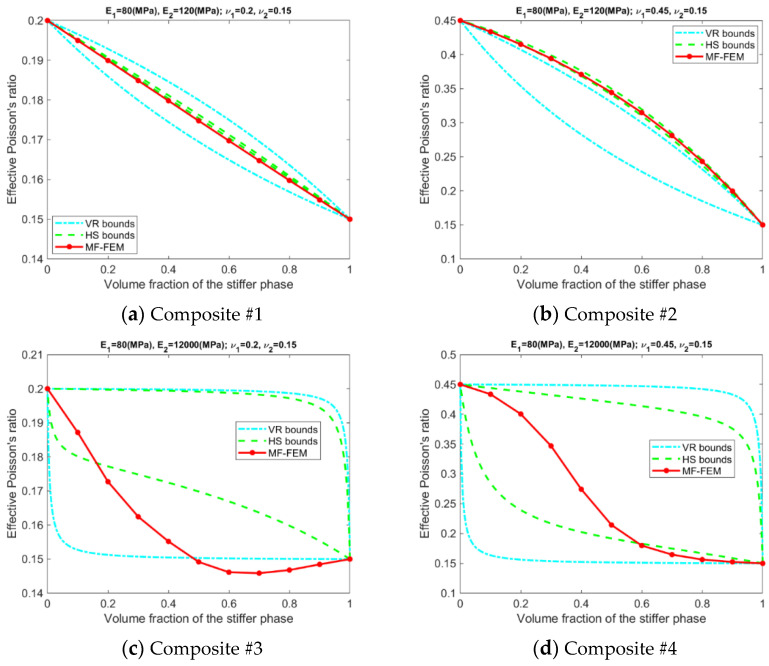
Relation between MF-FEM predictions and VR/HS bounds of effective Poisson’s ratio.

Results related to the VR and HS bounds are presented in Figure 3, Figure 4, Figure 5 and Figure 6. A number of phenomena can be detected from the results.

- Only if the contrasts of phase Young’s moduli and phase Poisson’s ratios are small, the VR and HS bounds are able to enclose the MF-FEM predictions.
- The gap between the upper and the lower bound of either HS or VR model is primarily dependent upon the contrast of phase Young’s moduli. If the contrast of phase Young’s moduli is small, the bounds are tight; otherwise, the bounds are loose. The contrast of phase Poisson’s ratios has a much lower significant effect on the gap.
- Contrary to the observations reported in some of the previous studies, the HS bounds may not be always enclosed by the VR bounds, e.g., the effective Young’s moduli in Figure 3b, the effective bulk moduli in Figure 5b, and the effective Poisson’s ratios in Figure 6b. This phenomenon is related to the large contrast of phase Poisson’s ratios.
- MF-FEM predictions may be out of both the HS and VR bounds, e.g., the effective Young’s moduli in Figure 3b and the effective Poisson’s ratios in Figure 6c.

## 4. Discussion

The results of this comparison study indicate that the differences between the predictions of MF-FEM and those of the selected micromechanics models are affected by both the contrasts of phase properties and the volume fractions; the largest differences occur in Composites #3 and #4, which have large contrasts in phase properties, particularly in phase Young’s moduli, and when the volume-fraction of the stiffer phase is high. Although previous experimental studies produced a large volume of data for composites that have small contrasts of phase properties and low volume-fractions of inclusions, e.g., [9,10,11,30,31] among many others, experimental data for composites that have large contrast and high volume-fraction of inclusions are rare in the literature for unknown reasons. Nevertheless, composites that have large phase contrast and high volume-fraction of inclusions indeed exist, bone is a good example. If bone is considered as an organic-inorganic composite [29], the organic and the inorganic phases have material properties of extraordinary disparity; nevertheless, the bone has superior mechanical properties over many engineering materials.

The rationality of using MF-FEM predictions in replacement of experimental data for the evaluation of micromechanics model accuracy is supported by a number of facts. First, MF-FEM predictions have good to excellent agreement with the experimental data produced from composites that have small phase contrast or from composites that have large phase contrast but low volume-fraction of inclusions [12]. Second, the MF-FEM is based on the same fundamental assumptions as the selected micromechanics models, that is, the composite is assumed statistically homogeneous and isotropic, and the material properties of the composite are independent of the shape and size of the inclusions. Theoretically, micromechanics models are more comparable with the MF-FEM than with mechanical testing, considering that the accuracy of both the MF-FEM and micromechanics models is only affected by their assumptions, while mechanical testing data may contain errors from different sources. To make analytical solution possible and simple, various special assumptions are adopted in the selected micromechanics models, which narrow the applicable scope of the models and result in low accuracy when the assumptions are not satisfied. The MF-FEM does not introduce any special assumptions. Therefore, it can be reasonably stated that the MF-FEM may not be able to completely replace mechanical testing, but the MF-FEM is more accurate than the micromechanics models. 

The differences between the MF-FEM and the selected micromechanics models are induced mainly by the special assumptions adopted in the models. For example, the assumption of dilute dispersion is explicitly or implicitly adopted in many micromechanics models including GSC and MT. By assuming that an inclusion is embedded in an infinite medium, dilute dispersion disregards the effect of interaction among inclusions. However, the rationality of this assumption disappears when the volume-fraction of inclusions is high. For composites that have small contrast of phase properties, the effect of inclusion interaction is trivial; for composites that have large phase contrast, the effect becomes much more significant. The dilute dispersion assumption is implicitly adopted in GSC and MT [32,33]. The limitations of conventional micromechanics models have been theoretically discussed in the literature [34,35,36,37]. The assumption of dilute dispersion is identified as the fundamental source for the limitations. The effect of dilute dispersion can only be fully revealed when the volume fraction is close to full packing of inclusions [3]. The Mori–Tanaka method is found unacceptably inaccurate when it is applied far beyond the usual dilute range [34]. Both VRH and Iso-VR are based on the iso-strain and iso-stress assumptions, which represent the two extreme scenarios that phase materials work together. Under the iso-strain condition, the phase materials work in parallel to achieve maximum stiffness, while under the iso-stress condition, the phase materials work in series to have maximum flexibility. The actual situation is somewhere between the two extreme scenarios, which may not have been accurately represented by either VRH or Iso-VR. The PEF formula was developed from bone mechanical testing data using statistical methods [29]. Although the formula is sometimes more accurate than the other models in the tested cases, the physical meaning of the formula is not as clear as the others. Another common issue for VRH, Iso-VR, and PEF is that they treat the four elasticity constants as independent parameters, but they are actually dependent upon each other. 

## 5. Conclusions

In this comparison study, the accuracy of the selected micromechanics models, including some of the most commonly used ones, is evaluated against the MF-FEM. The study results indicate that, only if the particulate composite has small contrasts in both of its phase Young’s moduli and phase Poisson’s ratios, the selected models have reasonable accuracy. When applied to composites that have large contrasts of phase properties, particularly the large contrast in phase Young’s moduli, the models may have extremely large error when the volume-fraction of the stiffer phase is high. The main limitation of the current study is that it is purely numerical based; an experimental study with the emerging 3D-printing technique would demonstrate the prospective application of the MF-FEM in engineering.

## Figures and Tables

**Table 1:** Two-phase particulate composites and their phase properties.

Composite #	Softer Phase		Stiffer Phase		Phase Contrast	
	Young’s Modulus (MPa)	Poisson’s Ratio	Young’s Modulus (MPa)	Poisson’s Ratio	Young’s Modulus	Poisson’s Ratio
1	80.0	0.20	120.0	0.15	Small	Small
2	80.0	0.45	120.0	0.15	Small	Large
3	80.0	0.20	12,000.0	0.15	Large	Small
4	80.0	0.45	12,000.0	0.15	Large	Large

**Table 2:** Symbols of composite and phase properties.

$E_{i}$	Young’s modulus of phase *i*		$\bar{E}$	Effective Young’s modulus of the composite
$G_{i}$	Shear modulus of phase *i*		$\bar{G}$	Effective shear modulus of the composite
$K_{i}$	Bulk modulus of phase *i*		$\bar{K}$	Effective bulk modulus of the composite
$\nu_{i}$	Poisson’s ratio of phase *i*		$\bar{\nu }$	Effective Poisson’s ratio of the composite
$f_{i}$	Volume fraction of phase *i*		$\bar{P},\;P_{i}$	Generic property of the composite and phase *i*

**Table 3:** RVE boundary conditions for the characterization of composite effective properties.

RVE Surface	Young’s Modulus ( ${\bar{E}}_{i},\;i=x,y,z$) and Poisson’s Ratio ( ${\bar{\nu }}_{ij},\;i,j=x,y,z$)		
	${\bar{E}}_{x}$ $,\;{\bar{\nu }}_{xy}$ $,\;{\bar{\nu }}_{xz}$	${\bar{E}}_{y}$ $,\;{\bar{\nu }}_{yx}$ $,\;{\bar{\nu }}_{yz}$	${\bar{E}}_{z}$ $,\;{\bar{\nu }}_{zx}$ $,\;{\bar{\nu }}_{zy}$
x=0	$u_{x}=0$	$u_{x}=0$	$u_{x}=0$
y=0	$u_{y}=0$	$u_{y}=0$	$u_{y}=0$
z=0	$u_{y}=0$	$u_{y}=0$	$u_{z}=0$
x=100	$u_{x}=1$	Homogeneous $u_{x}$	Homogeneous $u_{x}$
y=100	Homogeneous $u_{y}$	$u_{y}=1$	Homogeneous $u_{y}$
z=100	Homogeneous $u_{z}$	Homogeneous $u_{z}$	$u_{z}=1$

**Table 4:** Effective properties that are directly determined by MF-FEM and micromechanics models.

	Young’s Modulus ( $\bar{E}$)	Shear Modulus ( $\bar{G}$)	Bulk Modulus ( $\bar{K}$)	Poisson’s Ratio ( $\bar{\nu }$)
MF-FEM	x			x
VR bounds	x	x		
HS bounds		x	x	
VRH	x	x		
MT		x	x	
GSC		x	x	
Iso-VR	x	x		
PEF	x	x		

## Data Availability

Not applicable.

## Appendix A

Figure A1, Figure A2, Figure A3 and Figure A4 present predictions of the four effective properties, i.e., Young’s modulus, shear modulus, bulk modulus, and Poisson’s ratio, by the MF-FEM and by the selected micromechanics models for the four representative composites in Table 1, with the volume fraction of the stiffer phase varying from 0.0 to 1.0.

Figure A5, Figure A6, Figure A7 and Figure A8 show relative errors measured by Equation (13), in the effective Young’s modulus, shear modulus, bulk modulus, and Poisson’s ratio.
